# SMILES all around: structure to SMILES conversion for transition metal complexes

**DOI:** 10.1186/s13321-025-01008-1

**Published:** 2025-04-28

**Authors:** Maria H. Rasmussen, Magnus Strandgaard, Julius Seumer, Laura K. Hemmingsen, Angelo Frei, David Balcells, Jan H. Jensen

**Affiliations:** 1 Department of Chemistry, University of Copenhagen, Copenhagen, Denmark; 2 Department of Chemistry, University of York, York, UK; 3 Department of Chemistry, University of Oslo, Oslo, Norway

## Abstract

We present a method for creating RDKit-parsable SMILES for transition metal complexes (TMCs) based on xyz-coordinates and overall charge of the complex. This can be viewed as an extension to the program xyz2mol that does the same for organic molecules. The only dependency is RDKit, which makes it widely applicable. One thing that has been lacking when it comes to generating SMILES from structure for TMCs is an existing SMILES dataset to compare with. Therefore, sanity-checking a method has required manual work. Therefore, we also generate SMILES two other ways; one where ligand charges and TMC connectivity are based on natural bond orbital (NBO) analysis from density functional theory (DFT) calculations utilizing recent work by Kneiding et al. (Digit Discov 2: 618–633, 2023). Another one fixes SMILES available through the Cambridge Structural Database (CSD), making them parsable by RDKit. We compare these three different ways of obtaining SMILES for a subset of the CSD (tmQMg) and find >70% agreement for all three pairs. We utilize these SMILES to make simple molecular fingerprint (FP) and graph-based representations of the molecules to be used in the context of machine learning. Comparing with the graphs made by Kneiding et al. where nodes and edges are featurized with DFT properties, we find that depending on the target property (polarizability, HOMO-LUMO gap or dipole moment) the SMILES based representations can perform equally well. This makes them very suitable as baseline-models. Finally we present a dataset of 227k RDKit parsable SMILES for mononuclear TMCs in the CSD.

**Scientific contribution** We present a method that can create RDKit-parsable SMILES strings of transition metal complexes (TMCs) from Cartesian coordinates and use it to create a dataset of 227k TMC SMILES strings. The RDKit-parsability allows us to generate perform machine learning studies of TMC properties using ”standard” molecular representations such as fingerprints and 2D-graph convolution. We show that these relatively simple representations can perform quite well depending on the target property.

**Supplementary Information:**

The online version contains supplementary material available at 10.1186/s13321-025-01008-1.

## Introduction

SMILES strings and the associated molecular graphs are the foundation of cheminformatics and machine learning involving organic molecules. In addition to being a convenient way to share molecular data, SMILES strings that can be parsed into an RDKit mol object open the doors to the vast amount of cheminformatics tools available through RDKit [1]. For this reason RDKit has become a de facto standard within cheminformatics.

For transition metal complexes (TMCs), SMILES strings have a much less prominent role. Although it is possible to extract SMILES strings for TMCs in the Cambridge Structural Database (CSD [2]) only about half can even be parsed by RDKit.

The ability to go from a structure and overall charge to a SMILES/RDKit mol object is useful in various cases. As mentioned above, one important application is the ability to make full use of the data deposited in the CSD by enabling the tools included in RDKit. This includes tools for chemical alterations of the complex and for setting up quantum chemical calculations which is useful in screening applications. Also, using the SMILES in combination with RDKits similarity searching or fragmentation schemes could be a step towards a synthetic accessibility tool for TMCs. The task of obtaining SMILES/RDKit mol objects from a structure is complicated by the chemistry associated with TMCs, including the many possible oxidation states and bonding patterns for transition metals.

Previous work has been focused on extracting relevant molecular data for TMCs from the CSD. Vela et al. developed *cell2mol* [3] which extracts information from the crystallographic data in the CSD needed to do quantum chemical calculations. This includes the connectivity and total charge of the molecules in the unit cell as well as the oxidation state of the metal. For a dataset consisting of mono-metallic species with eight different transition metals they were able to interpret 77% of them (with 95% of the interpretations being correct).

Balcells and coworkers. have done extensive work to make TMC datasets from the CSD available [4–6]. This includes graph representations of 60k mono-metallic closed shell TMCs with an overall charge of −1, 0 or 1 (tmQMg) [5]. The connectivity in these graphs are based on natural bond orbital (NBO) analysis from density functional theory (DFT) calculations. Recently, Kneiding et al. extended this dataset with charge information for 30k ligands present in the TMCs of tmQMg also based on a NBO analysis (tmQMg-L) [6].

The combined work of Balcells and coworkers really became a turning point when it comes to generating TMC SMILES from structure. Based on the connectivity and ligand charges one can use a program such as xyz2mol to generate RDKit mol objects for the TMCs [7]. Here we make RDKit parsable SMILES based on the NBO connectivity and ligand charges from DFT calculations [5, 6]. To get an idea of the quality of these SMILES, we compare these SMILES to the SMILES available through the CSD. However, as mentioned above these SMILES tend to not be parsable by RDKit. Therefore we put these SMILES through a series of fixing-steps in order to get a sanity check of the obtained SMILES.

In principle the above method, based on NBO analysis of a DFT calculation followed by a xyz2mol-based workflow for assigning formal charges and bond orders, represents a method for obtaining SMILES/RDKit mol objects based on structure and overall charge. However, relying on DFT calculations makes this method very slow, limiting its use cases. Therefore we propose a much faster approach based on atomic distances and extended Hückel calculations (available through RDKit) for the ligands [8]. We will show that this is a robust way of obtaining a SMILES representation of a TMC that can be read into an RDKit mol object. Furthermore, we can assess the quality of this representation by comparison to the fixed CSD SMILES as well as the SMILES created based on the DFT-NBO analysis for tmQMg and tmQMg-L [5, 6].

Kneiding et al. utilized their graph-based dataset (tmQMg) to perform different machine learning tasks using graph convolutional neural networks (GCNNs) [5]. Since SMILES strings represent a 2D molecular graph, these can readily be used to create graphs for GCNNs. Furthermore, since the SMILES can be read into RDKit mol objects, the nodes and edges can be featurized using RDKit and RDKit offers several fingerprint featurization schemes. Molecular fingerprint featurizations such as ECFP4 [9] represent a standard withing machine learning for organic chemistry and often performs well for smaller datasets [10]. We demonstrate the performance of these RDKit-based graphs as well as ECFP4 and compare to the DFT-NBO based graphs from Kneiding et al. For a 3D property such as the dipole moment, we find that the models fed with QM and 3D informed graphs perform significantly better than the models based solely on information available through SMILES which are 2D by nature. However, for the HOMO-LUMO gap and polarizability the RDKit-based graphs perform close to on par with the QM/3D informed graphs.

## Computational methodology

Below we describe our three approaches for obtaining SMILES for TMCs; fixing CSD SMILES, using xyz2mol with DFT-NBO input and xyz2mol with extended Hückel input. The latter requires only the xyz-coordinates, overall charge and RDKit, whereas the other two require either an initial SMILES from the CSD or a DFT calculation yielding the necessary NBO data. Since TMC SMILES are not currently incorporated in chemical research, there is no established consensus on how to represent e.g. bonds to transition metals. Here we choose a representation, where the electrons in the TM-ligand bond are located on the ligand, i.e. all TM-ligand bonds are represented by dative bonds (symbolized by an arrow in the SMILES). This has the advantage of making the ligand charges and metal oxidation states directly available.

**Fig. 1:**
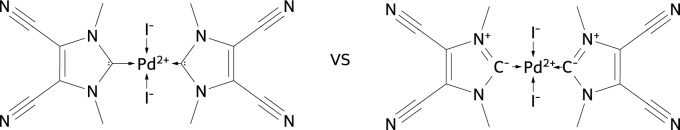
We choose the carbene representation (left hand side) over the charged representation (right hand side) when either solution is possible. CSD ID: BOCPEP

Contrary to organic chemistry relating to e.g. drug discovery, a chemical entity such as a carbene is a lot more common as a ligand in a TMC. Often another representation obeying the octet rule could also be drawn (Fig. 1). Here, we choose the carbene representation (non-zwitterionic). The carbene representation has the advantage of avoiding the ambiguity of assigning the positive charge on nitrogen for asymmetric ligands.

### SMILES string from extended Hückel and distance data

**Fig. 2:**
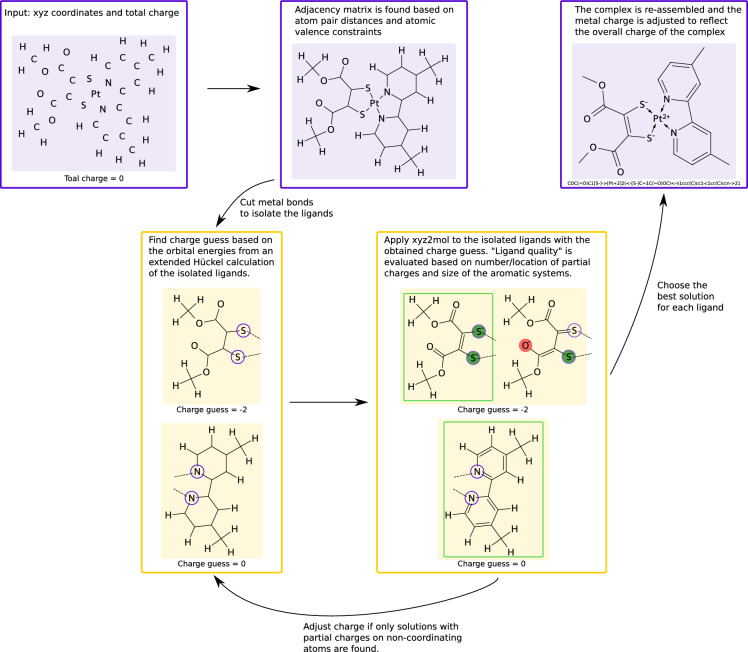
The major steps involved in the xyz2mol procedure for TMCs

The SMILES generated from DFT-NBO data are based on high quality (and expensive) calculations of both ligand charge and bonding pattern. In contrast, using an extended Hückel calculation for the charge guess and a simple distance cutoff for the bonding pattern can be expected to make wrong predictions frequently. Thus, when working with extended Hückel calculations we apply a custom procedure to catch and possibly correct these wrong predictions. The procedure going from xyz coordinates and overall charge (*Q*) to an RDKit mol object is outlined in Fig. 2 and is as follows.

- An initial adjacency matrix (AC) is calculated based on the xyz-coordinates. An AC is an $N_{\text {atom}} \times N_{\text {atom}}$ matrix where $N_{\text {atom}}$ is the number of atoms in the molecule. An element, AC$_{\text {ij}}$, is 1 if atom i and j are bound and 0 otherwise. Following Open Babel [11], a bond (one in the AC) is created if the distance between two atoms is shorter than the sum of their covalent radii plus a tolerance of 0.45Å. Since this can create valence violations (e.g. five bonds to carbon), a subsequent filter is applied cutting the weakest bond to an atom until the valence requirements are met. Here, the weakest bond is defined as the longest bond relative to the covalent radii of the atoms. More exotic bonding environments such as bridging hydrogen atoms can thus not be described using the default settings since this would violate the maximum valence of hydrogen which is one.
- The ligands are identified based on the AC and an extended Hückel calculation is done for each isolated ligand using RDKit [1, 8]. An initial guess for the ligand charge is calculated as
  1 $$\begin{aligned} q = \sum _{i}^{N_{atom}}V_i - \sum _k^{E(\psi _k) < E_C}2 \end{aligned}$$
  where $V_i$ is the number of valence electrons for atom *i*, {$\psi _k$} is the set of molecular orbitals generated with extended Hückel theory, $E(\psi _k)$ is the energy of orbital *k* and $E_C = 10$ eV is the cutoff energy under which the molecular orbitals are filled with double occupancy. If this results in a positive ligand charge combined with a low lying LUMO ($E(\psi _{LUMO} < -9$ eV), we iteratively add two electrons until this is no longer the case. Similarly, if this results in a ligand charge of −2 or lower combined with a high lying HOMO ($E(\psi _{HOMO})> -10.2$ eV), we iteratively remove two electrons until either of the two criteria are no longer true.
- Using xyz2mol [7], an RDKit mol object is generated for the ligand using the charge guess. This is done by looping over all combinations of allowed valences for the atoms until a solution matching the required charge and ligand AC is found. If no sanitizable solution is found, the charge is adjusted; if $q \ge 0$ we add two electrons, otherwise we remove two electrons. If still no sanitizable solution can be found no SMILES is generated.
- If a sanitizable mol object is generated, we proceed to check different resonance forms of the ligand and choose one based on the following criteria: (1) A larger aromatic system is preferred over a smaller one. (2) Fewer formal atomic charges are preferred, but negative charges on the metal-coordinating atoms of the ligand do not count against that resonance form.
- The above two steps are repeated with settings disallowing carbenes and the found resonance forms are evaluated based on the same criteria. Finally the best found ligand representation from either this or the previous step of each ligand is kept.
- The oxidation state of the metal ($M_{ox}$) is calculated from the overall charge and found ligand charges ($q_i$): $M_{ox} = Q - \sum _i^{N_{\text {lig}}}q_i$.
- The complex is re-assembled based on the initially found AC. All metal-ligand bonds are represented with dative bonds.
- Finally a couple of sanitization steps (described below) are performed to catch known problematic behavior before a SMILES is generated for the mol object of the complex using RDKit.

#### Cleaning steps

**Fig. 3:**
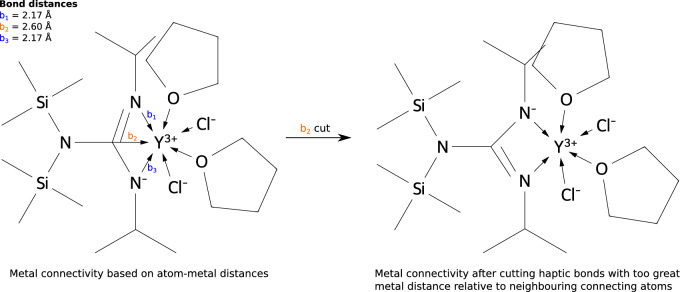
Example of cleaning procedure for the metal coordination environment. CSD ID : PIYNER

Setting valence constraints for the atoms involved in simple organic molecules is fairly straightforward; carbon and nitrogen should not have more than four bound neighbors, hydrogen and fluorine can only have one bound neighbor and so on. Defining valence constraints for transition metals is a lot more complicated. Initially, the AC of the complex is found based on atom pair distances as described above. The valence constraints are invoked based on the non-TM atoms, cutting the longest bond relative to the atomic radii of the atoms involved in the bond if a ligand atom has too many neighbors. In some cases, this leads to a “fake” haptic bonding pattern i.e. a situation where the neighbor-atom of a coordinating atom is initially also assigned a bond to the transition metal even though the distance to the TM is significantly larger (Fig. 3). We avoid this by cutting perceived haptic bonds that are much longer than those for their haptic neighbors.

**Fig. 4:**
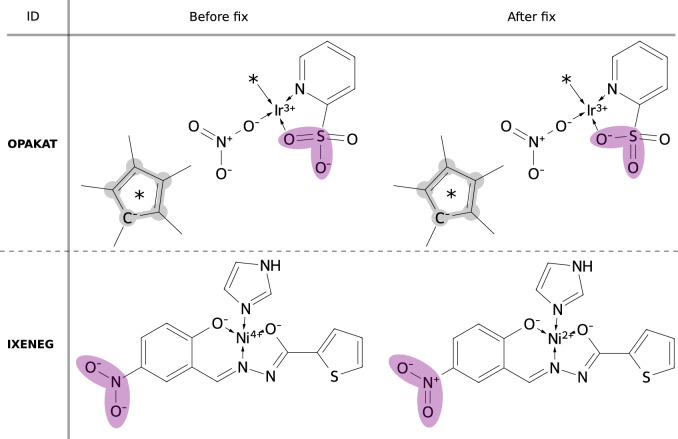
Example of moving a negative charge due to ligand atoms being equivalent for the isolated ligand but not for the whole complex (OPAKAT). IXENEG is an example of the NO$_2$-group fix: we make sure all NO$_2$-groups are described in the neutral form

While the above described loop through stereoisomers of the ligand to find the best atomic charge distribution works well in most cases, there are a few examples where the negative charge is in fact not assigned to the coordinating atom. The sulfonate anion in OPAKAT (Fig. 4) is an example of the negative charge initially not being assigned to the coordinating atom. For the isolated ligand, the oxygen atoms are equivalent and only one resonance form with the charge assigned to a random oxygen is created. This is one of two post SMILES creation fixes that are done using SMARTS pattern matching. The other post fix relates to –NO$_2$ groups that we want to consistently be described with the same representation. Two Lewis structures obeying the octet rule can be drawn for an –NO$_2$ group (IXENEG, Fig. 4), both with two formal charges. We make sure to always use the neutral representation, adjusting the metal oxidation state accordingly.

### SMILES strings from DFT-NBO data

Kneiding et al. [6] provide the tmQMg-L dataset of 30 K TMC ligand charges, metal coordination atoms and their corresponding 3D coordinates. Using this data in combination with xyz2mol we create TMC SMILES representations of the TMCs in tmQMg. This is done by first using xyz2mol to obtain SMILES strings for the isolated ligands in tmQMg-L and then combining them with a TM to form the full tmQMg TMCs. The approach is described in the following text.

- Knowing the ligand composition of a complex, we attempt to generate RDKit mol objects for each ligand in the complex. The xyz2mol approach for the ligands is the same as the extended Hückel approach explained above except that the ligand charge is fixed.
- If mol objects can be obtained for all ligands in a TMC, the coordinating atoms for each ligand are connected through dative bonds to the TM for the given TMC. The 3D coordinates of the ligands are given by the ligand xyz files and the coordinates of the core TM are parsed from the TMC xyz file.
- We then set the metal formal oxidation state by subtracting the sum of the ligand charges from the TMC overall charge. In this way the overall charge of each TMC SMILES will match the tmQMg dataset. However, TM formal oxidation states can still be inaccurate compared to the true CSD structure if ligand charges from the DFT-NBOs are wrong.
- In the final step we sanitize the RDKit mol object and remove the hydrogens. Complexes that fail this procedure likely have wrong ligand charges or incorrect coordination atoms and therefore do not get a SMILES.

### SMILES strings from the CSD

SMILES strings of mononuclear TMCs were extracted from the CSD as described by Frei and Orsi [12] except that only d-block elements and all coordination numbers were considered. The initial screening process eliminated cases where the counter-ion also contained a metal, which eliminated some compounds that were included by Balcells and co-workers. We add these entries, making tmQMg a subset of the CSD dataset, and remove complexes containing group 1 or group 2 elements and complexes containing > 100 heavy atoms. This results in 230,550 SMILES. For 7297 TMCs we are unable to retrieve a SMILES via the API and 7 entries have been deleted from the CSD after tmQMg was curated. Of the remaining 223,246 SMILES, RDKit can only successfully sanitize 123,147 SMILES strings and we therefore implemented a custom SMILES fixer process that all CSD SMILES strings were subjected to.

The two main reasons that RDKit cannot sanitize the SMILES are (1) failure to kekularize aromatic rings and (2) improper valences such as a N atom with four bonds lacking a positive charge. The kekularization error always involves at least one ligand atom covalently bonded to the metal (the CSD SMILES do not contain dative ligand-metal bonds), while this is not necessarily the case for atoms with improper valences. The cleanup process involves the conversion of all ligand-metal bonds to dative bonds. If the bond is judged to be a “true” covalent bond, based on its valency, then the charge on the metal and the ligand atom is increased and decreased, respectively, according to the bond order. For example, if the bond order is one, then the charge of the metal atom and ligand atom is increased and decreased by one unit, respectively. Occasionally, the CSD SMILES string does not contain the explicit hydrogen atoms commensurate with the bonding type, leading to a wrong assignment of atomic charges. However, these errors are typically caught (though not corrected) since such errors will lead to the wrong number of implicit hydrogen atoms being added in the sanitation step, which in turn, leads to the wrong empirical formula of the compound. Atoms with incorrect valencies (typically N and B atoms) that are not directly bonded to the metal have formal charges assigned in a similar way. A +1 charge is assigned to hypervalent N atoms while the charge of the metal is decreased by one unit, while the opposite is true for hypervalent B atoms. In addition, SMARTS-based fixes are done for special cases like carbenes, carbon monoxide, metal triple bonds to O and N atoms, and pyrrole.

All in all, these changes allow for 213,278 SMILES to be sanitized. 53,344 of which are part of the tmQMg dataset.

### Property prediction from SMILES

We use the obtained SMILES sets to perform the property prediction tasks as done by Kneiding et al. [5] where we predict polarizability, HOMO-LUMO gap and dipole moment for the tmQMg complexes using our TMC SMILES.

To encode the SMILES we apply both fingerprint and graph representations. For the fingerprint representation we use Morgan count fingerprints with radius 2 (ECFP4) and bit size 1028 [9, 13]. For the graph representation we use an RDKit graph featurization approach that constructs node and edge features from the information available in the SMILES strings [14].

For consistent comparison with [5] we construct our test set to match theirs. We create a test set that contains the maximum overlap between complexes with valid SMILES and the complexes used in their original test set of 5615 complexes. The Hückel SMILES set has the largest overlap with 5534 SMILES. As we observe the same trends for all three SMILES sets, the discussion is limited to the ML models trained on the Hückel SMILES. To ensure consistent comparison we also re-evaluate the models from Kneiding et al. on this smaller test set after re-training on the slightly larger training set. The models from Kneiding et al. [5] are referred to as Baseline, u-NatQ, d-NatQ. Here, the Baseline model is trained on graphs informed with standard atom and bond properties. The u-NatQ and d-NatQ models are trained on graphs informed with NBO topology and attribution. u-NatQ on undirected graphs and d-NatQ on directed graphs.

We test the performance of the fingerprint and graph representations with five different models. Namely, Random Forest (RF), Feed-Forward Neural Network (FF-NN), light gradient boosting machine (LightGBM [15]), a simple Graph Convolutional Neural Network (GCNN) and the Message Passing Neural Network (MPNN) by Gilmer et al. [16]. The Gilmer MPNN is identical to the one used by Kneiding et al. [5], the difference being that we featurize our graph representations based on RDKit properties that can be obtained directly from our SMILES. For additional details on ML training see section S1 in the SI.

## Results

### Comparing SMILES sets

**Fig. 5:**
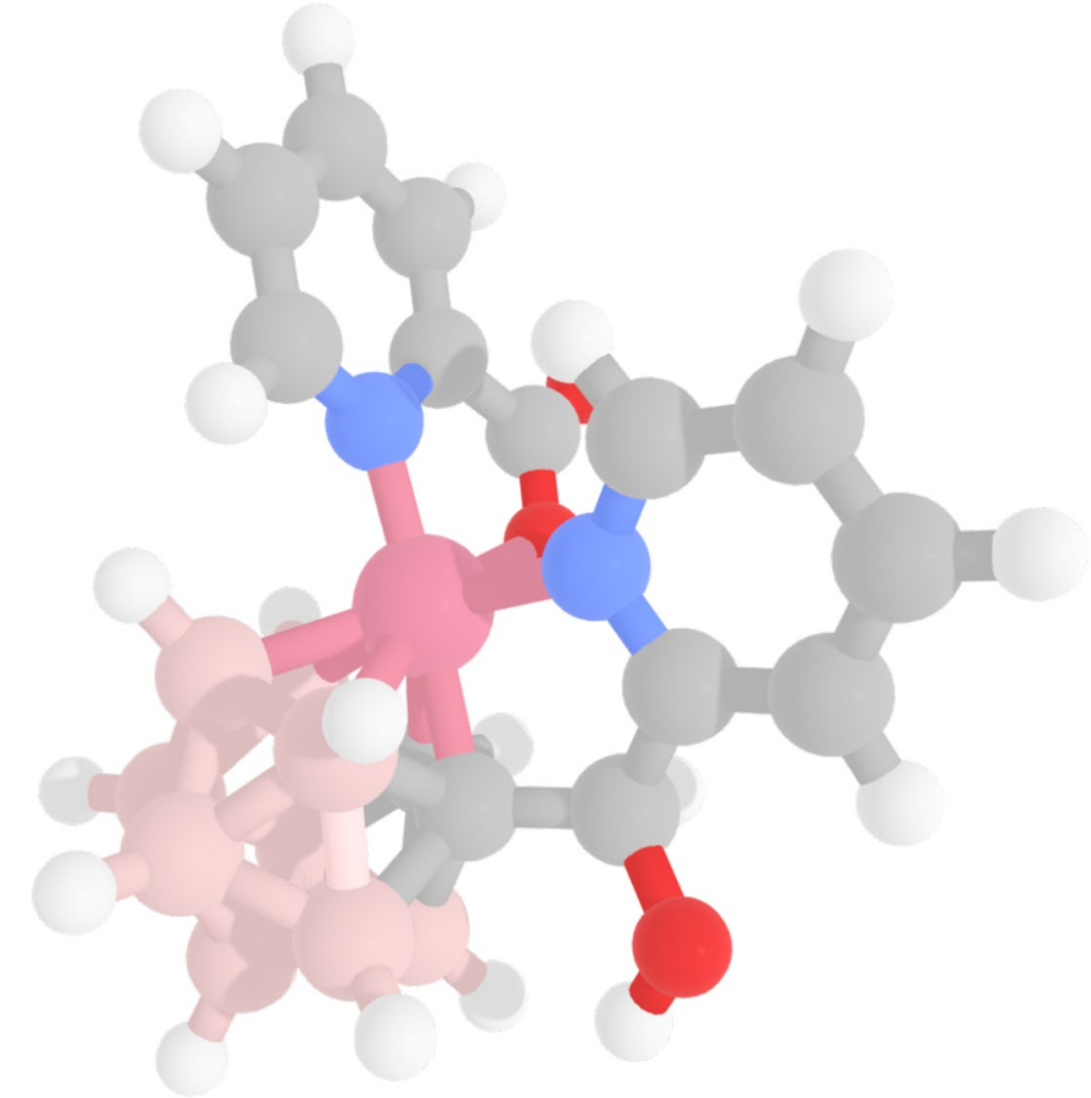
Example of a molecule containing a boron hydride cluster. None of the above described methods can produce a SMILES/mol object for these kind of structures due to the high valence of Boron. CSD ID: PEKGAP. Dark pink: Cobalt, light pink: Boron, red: Oxygen, blue: Nitrogen, grey: Carbon, white: Hydrogen

The tmQMg dataset consists of 60,799 TMCs with both the deposited CSD structure and a DFT optimized structure available. For the DFT/NBO method we are able obtain 46,190 valid RDKit parsable SMILES. For the Hückel/distance method we are able to obtain 59,878 valid RDKit parsable SMILES using the DFT optimized structures and 59,893 RDKit parsable SMILES using the CSD deposited structures. Lastly, using the sanitization procedure for the CSD SMILES we obtain 53,320 RDKit parsable SMILES. For the Hückel SMILES, the majority of the cases where we do not obtain a SMILES contain some kind of boron hydride structure (Fig. 5). Proper description of these complexes requires 3-center 2-electron bonds, which is not currently possible and these complexes will generally fail in all approaches due to the high valence of boron. The DFT/NBO method returned the lowest number of SMILES which is mostly due to all necessary ligand information not being available for $\approx$ 8k complexes. As stated by Kneiding et al. [6] some ligands are discarded based on the results of the DFT calculations. For the remaining complexes, a solution could not be found that would satisfy the given AC, ligand charges and possible atomic valence combinations. Approximately 4k of the complexes in the tmQMg dataset did not have a SMILES available from the CSD API and thus cannot be run through our fixing procedure. The remaining $\approx$ 4k SMILES missing are from mol objects that cannot be sanitized even after the above described fixes have been implemented.

**Fig. 6:**
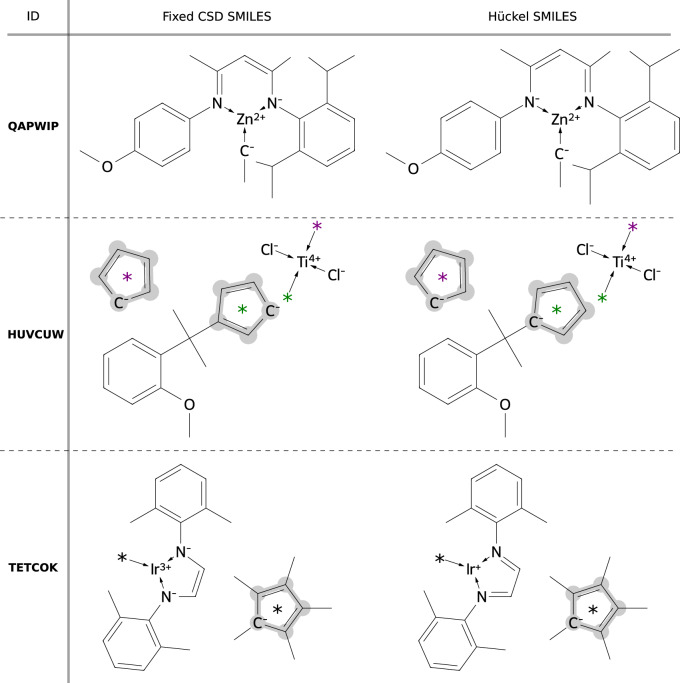
Examples of fixed CSD SMILES compared to Hückel SMILES where both representations are valid. For QAPWIP and HUVCUW, the SMILES are resonance forms of each other. The two solutions for TETCOK differ in their ligand charge (and hence oxidation state of Iridium). However, one solution is not clearly superior to the other

For 41,355 complexes, we obtain a SMILES from each of the three methods. 29,053 of these (corresponding to 70 %) are completely identical across all three methods. These SMILES can be assigned a high level of confidence. When the SMILES do not exactly match it can be due to several things. The AC of the complex can differ, resulting in different SMILES. Most frequently the difference is in the metal-ligand bonds. Another possibility is that the ligand charges (and hence oxidation state of the metal) differ. For some ligands, multiple total ligand charges can result in chemically reasonable charge configurations (see e.g. TETCOK in Fig. 6). Thus, lack of agreement between SMILES does not necessarily mean that one (or all) of the SMILES are bad, but can also mean that multiple reasonable representations exist. Even if the ligand charges and AC matrix of the complex are identical across methods, the obtained SMILES can still be different due to different resonance forms. QAPWIP and HUVCUW (Fig. 6) are examples where the SMILES obtained from the fixed CSD SMILES approach and the Hückel approach are resonance forms of each other.

In Table 1 we compare the three methods in pairs. Direct comparison of the SMILES results in >70% match for each of the three pairs. The pair with the highest percent-wise overlap is found for NBO(DFT)/Hückel, where 81% of the SMILES match. Considering only the highest amount of matching SMILES we find the CSD/Hückel pair to be the largest with 38,073 SMILES. Considering that these two methods of obtaining SMILES are completely different, these 38,073 SMILES can be assigned a high level confidence. When comparing the resonance forms of the same TMCs the number of equal SMILES increases only slightly across the three pairs. Finally, we compare the resonance forms after disconnecting the ligands from the TM (Disconnect-Resonance). This increases the number of SMILES matching with between 5k and 7k. These TMCs differ only in the coordination to the TM (and possibly ligand resonance structure) but have identical ligand charges and TM oxidation state.

For the SMILES that do not match, a plethora of reasons can exist. Some cases resemble that of TETCOK (Fig. 6) but others are more problematic with e.g. a disconnected graph due to valence constraints. We analyze some of these cases in Sect. 3 and show various examples in https://github.com/jensengroup/xyz2mol_tm/blob/main/comparing_smiles/highlight_smiles_problems.ipynb.

It is a known issue that for some 3D structures in the CSD, the 3D structure does not necessarily reflect the expected structure. For some structures here are hydrogens missing in the 3D structure. Additionally, the fixed CSD SMILES strings can have incorrect number of implicit hydrogens due to the nature of the fixing procedure. Both these cases can be detected by comparing the formula of our SMILES with the corresponding formula from the CSD API. How this is detected and for which complexes this is the case is shown in the notebook mentioned above.

**Table 1:** Number of equal SMILES when comparing the three SMILES pairs with different methods

Method	SMILES pair		
	CSD/NBO(DFT)	CSD/Hückel	NBO(DFT)/Hückel
Direct comparison	31 675/41 432	38 073/53 167	37 500/46 103
Resonance TMC	31 929/41 432	38 442/53 167	37 565/46 103
Disconnect-Resonance	37 215/41 432	45 734/53 167	42 468/46 103

Direct comparison compares the canonical SMILES, Resonance TMC compares the SMILES resonance sets and Disconnect-Resonance compares the resonances of ligands when disconnected from the TM

### SMILES distributions

**Fig. 7:**
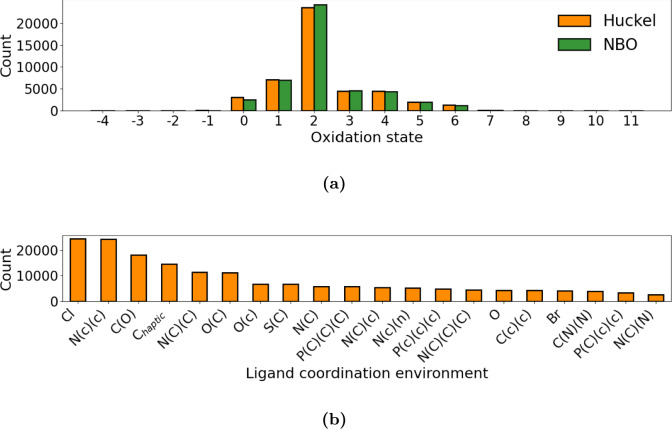
**a** TM oxidation state distribution of the Hückel and NBO SMILES. **b** Top occurring coordination environments in the 58 K Hückel SMILES. The first atom is the coordination atom and the following atom labels enclosed by parenthesis are the direct neighbors to the coordinating atom. Lower case signifies an aromatic atom

With a SMILES dataset of the TMCs present in the CSD, one can easily access various information of the TMCs. This includes key attributes of TMCs such as the oxidation state of the TM metal and the ligand coordination environments. We show the oxidation state of the TM for TMCs where we have valid SMILES for both the NBO and Hückel methods in Fig 7a. Figure 7b shows the TMC ligand coordination environments for the Hückel SMILES.

We observe that the TMCs in the tmQMg contain a wide range of oxidation states. The majority of which lies in the range 0–6 with oxidation state +2 being the most common with around 25 K occurrences. A small portion of the SMILES have TMs with oxidation states that are unusually low ($< 0$) or unusually high ($>10$). Negative oxidation states are not unheard of [17] but the occurrence of oxidation states $>10$ is clearly an artifact of the method since the highest oxidation state found in a stable TMC is 10 [18]. For the Hückel method a total of 26 TMCs are described with a formal oxidation state > 10. Almost all of these complexes contain the perchlorate ion, i.e. a chlorine atom bound to 4 oxygen atoms, which is violating our set maximum valence for chlorine. Since the AC is generated by cutting bonds until the maximum valence is complied with, this results in a lot of lonely oxygen atoms, which are assigned a charge of −2. This in turn makes the oxidation state of the TM artificially high (Fig. 3). Depending on the type of TM different oxidation states will be impossible or at least unusual. It is likely that most of the SMILES at the extrema are non accurate representations of the TMCs as a result of the method used as in the case of the perchlorate ion. Such extreme cases are highlighted in Sect. 3.

Furthermore, the Hückel and NBO SMILES have similar distributions of the oxidation states. A larger discrepancy between the two methods is observed for oxidation states 0 and +2. This is mainly attributed to the difference in orbitals between the NBO and Hückel methods. For the NBO method 1166 ligands are attributed a −2 charge while they are assigned a 0 charge with the Hückel method. Conversely, 262 ligands are attributed a −2 charge with Hückel where the NBO method gives a 0. As such, this results in a slightly higher peak of +2 oxidation states for the NBO method. Examples of this are shown in Sect. 3.

Figure 7b shows the importance of chloride, pyridine and carbonyl coordination in TMC chemistry. Here we leverage the graph information in the SMILES to extract these coordination patterns that also includes information on aromaticity of the coordinating atoms neighbors. Such insights are crucial when making design choices in accelerated TMC discovery as they can be used to adjust the search scope or to design custom synthetic accessibility (SA) scores for use in generative models. An example would be in TMC catalyst discovery where ligand coordination environments can be used to either set up a starting geometry for TMCs to search for, or as a look-up table for potential coordination environments in new ligand candidates.

### ML with TMC SMILES strings

**Fig. 8:**
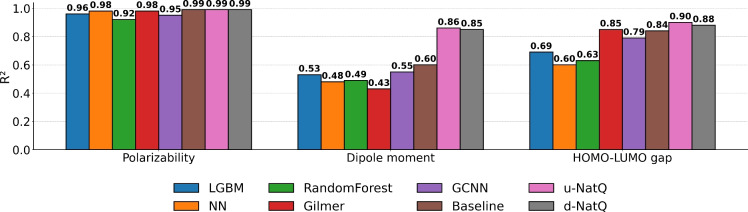
Comparison of $R^{2}$ values for the 5 SMILES based models versus the 3 DFT-NBO based models for the test set

We train a range of model for the tmQMg dataset based on the Hückel SMILES. This was the method yielding the highest number of SMILES as well as the most general method since it requires neither DFT calculation and NBO analysis nor an initial SMILES representation. The LightGBM, Random Forest and FF-NN models are trained on the ECFP4 with a bit vector length of 1024. The GCNN and Gilmer models are trained on graph representations generated from information inherent in the SMILES. Finally, the Baseline, u-NatQ, d-NatQ models are trained on the NBO graph data from [5]. Note that the u-NatQ and d-NatQ graphs are fed with node and edge information extracted from DFT calculations. Thus, when generating these graphs the target property (polarizability, HOMO-LUMO gap or dipole moment) is immediately available. In addition, the baseline, u-NatQ and d-NatQ graphs contain some 3D information as it has bond lengths as an edge feature from the optimized geometry. Contrary to this, the models based on SMILES only have the 2D graph defined by the SMILES, as well as the cheminformatics properties available through RDKit. All models are evaluated on the same test set as outlined in the computational methodology. We apply a naive approach where all remaining SMILES in the Hückel SMILES set are used for training. A comparison of the resulting $R^{2}$ correlation metrics are seen in Fig. 8.

Generally, there appears to be no difference among the methods when it comes to predicting polarizability. The polarizability is to a certain extent an additive property. As such, this correlation can be easily learned with simple ML models based on circular fingerprints with little to no gain in using more advanced models and/or molecular featurizations.

For the HOMO-LUMO gap a larger discrepancy is apparent between the performance on the training and test sets. Surprisingly, the Gilmer model trained on the SMILES graph data matches the performance of the Gilmer models trained on the significantly more expensive DFT-NBO data. The GCNN also performs much better than the fingerprint models.

Finally, the hardest task for the models is the dipole moment. Again, the models struggle to extrapolate to the structures in the test set. Here, the Gilmer and GCNN models struggle to learn the dipole moment from the SMILES graphs which is due to the fact that the dipole moment is instrisically a geometry dependent property. Since the Baseline, u-NatQ, and d-NatQ models include bond distances it enables the model to learn the correlation between geometric bond information and the dipole moment. For comparison, the baseline model without bond distances as edge features get an $R^2$ value of 0.42 (this value was kindly offered by the authors). This is something that one can not obtain from only the 2D graphs defined by the SMILES strings. Note that preliminary results predicting dipole moments for organic molecules using ECFP4 showed equally bad results.

One possible remedy for this is to enrich the SMILES with chiral tags reflecting the stereochemistry of the metal center. This information is available through the TMC 3D coordinates and it is possible to specify the geometry of a TMC and the arrangement of the ligands around the metal which could aid the model in learning geometry dependent properties like the dipole moment. However, in the current state of RDKit (2024.03.3) this functionality is unreliable and it was therefore not possible to incorporate stereochemistry of the TM into the SMILES. More details are outlined in S4.

### A SMILES data set of 227k TMCs from the CSD

As mentioned above, a total of 230,550 mononuclear TMCs were extracted from the CSD and as many of their available SMILES as possible were made sanitize-able for a total of 213,278. Using the Hückel method, we obtain SMILES for 217,776 of the 230,550 extracted TMCs. Combining these two methods means that we make 227,124 SMILES of CSD TMCs, that can readily be read into RDKit, available. For those complexes where we have a SMILES for both the fixed CSD and Hückel methods we compare the SMILES in Table 2.

We observe that 69% of the SMILES are identical as is, with an increase to 76% when also considering TMC resonance forms and finally 79% when disconnecting the ligands. Following the analysis for Table 1 the SMILES that are equal for both methods can be assigned a high level of confidence. Furthermore, the fraction of identical SMILES does not deteriorate when looking at the larger CSD set which highlights that the Hückel method is not only effective at the smaller tmQMg subset.

When using this dataset, e.g. for machine learning, the conservative choice is to only use the “fixed” CSD SMILES since the connectivity is determined, at least in parts, by experts. Bond perception presents the biggest challenge for xyz2mol and most of the SMILES-differences is due to differences in connectivity. That being said, differences in connectivity could possibly be an indication of weaker coordination, so there may be a benefit to using both SMILES in an ML project as a form of data augmentation, for cases where coordination strength is important. Similar considerations also apply in cases where the resonance forms or oxidation states are ambiguous (i.e. differ).

**Table 2:** Comparing the CSD fixed SMILES set with the corresponding Hückel SMILES for the large 230 K set dataset

Method	SMILES pair
	CSD/Hückel(CSD)
Direct comparison	140 510/203 930
Resonance TMC	154 354/203 930
Disconnect-Resonance	161 041/203 930

Direct comparison compares the canonical SMILES, Resonance TMC compares the SMILES resonance sets and Disconnect-Resonance compares the resonances of ligands when disconnected from the TM

## Discussion/conclusion

We present an xyz2mol method for TMCs. Using only an xyz file and an overall charge we are able to obtain TMC SMILES that contain atomic charges and TM oxidation states. We use this method to provide two large datasets of TMC SMILES for TMCs found in the CSD. The first dataset consists of 3 different SMILES sets for complexes in tmQMg (a subset of mononuclear TMCs in the CSD with charge −1, 0 or 1 developed by Kneiding et al. [5]) obtained with 3 different methods where our Hückel approach was able to obtain SMILES for 59 878 of the complexes in the tmQMg. At least 38,442 of these SMILES are expected to be highly reliable and acurate as they are identical with those from the CSD after the fixing steps to make it sanitizable in RDKit. The matching percentage between xyz2mol SMILES based on Hückel calculations and CSD SMILES is 72%. For comparison, xyz2mol SMILES based on the high quality (and expensive) DFT-NBO data get a similar matching percentage of 76%. Note that this percentage is not including the $\approx$ 14k complexes where a SMILES could not be obtained for the DFT-NBO method.

The second dataset consists of 213,278 SMILES from the CSD which are RDKit parsable as well as 217,776 SMILES from the Hückel method. In total we present RDKit parsable SMILES for 227,124 mononuclear TMCs from the CSD using either the CSD fixing method or the Hückel method.

These SMILES sets can be used to train baseline ML models serving as a reference for more elaborate methods. We show that using our SMILES to obtain fingerprints or graphs and then training ML models on these representations, we are able to predict polarizability and HOMO-LUMO gap to the same level of accuracy as Kneiding et al. [5]. As such, the SMILES graphs are sufficient for this task, avoiding the need for expensive DFT-NBO calculations. The lack of 3D information in the SMILES hinders the accurate prediction of the dipole moment and we are therefore not able to match the performance of the DFT-NBO data. This performance and the fact that the SMILES are not filtered for inaccurate representations, combined with the ease of which the SMILES can be obtained with the Hückel method, illustrates how powerful these representations can be for TMC development.

One thing we have been missing in our own research on catalyst optimization for TMCs is a synthetic accessibility (SA) score that works for TMCs [19–21]. So far we have applied a synthetic accessibility score designed for drug-like molecules for the isolated ligands which steers the proposed ligands to look like drug-like molecules [22, 23]. A similar score for TMCs would clearly be desirable for generative models proposing new TMCs. This SMILES-based dataset of the CSD serves as a good starting point of generating such a SA score. Kerstjens et al. recently proposed a method for correcting molecules from generative models based on a “familiarity” concept that takes into account which atom/bond types as well as fragments are present in a reference library [24]. While they applied it to a reference library consisting of drug-like molecules we are currently applying it to our CSD SMILES dataset since the method requires molecules to be parsable by RDKit to get a score for whether a new TMC is “familiar” to the CSD.

While we have tried to retain a large amount of flexibility when it comes to what chemistry can be described, i.e. which atom types and valences are allowed, there is still examples of chemistry that we are currently incapable of describing properly. This includes the examples highlighted in the manuscript such as boron hydride clusters and the perchlorate ion. Future work will include expanding the chemistry that can be described while retaining and improving the robustness and reliability of the Hückel method. One possible improvement is to take into account the preferred oxidation states of the TMs. Especially in cases where multiple ligand charges result in chemically reasonable structures, one could let the TM decide which to choose based on a prioritized list of oxidation states. Other improvements would involve proper logging of potential issues like unusual TM oxidation states or uncommon ligand charges or coordination environments. We expect that how the community will use this tool will guide the direction of improvement, when undoubtedly problematic cases that we have not thought of arise.

## Supplementary Information

Supplementary Material 1.

## Data Availability

All code used to create the TMC SMILES can be found in the designated code repository: github.com/jensengroup/xyz2mol_tm This includes the script for the Hückel to SMILES approach found at xyz2mol_tm/huckel_to_smiles/xyz2mol_tmc.py The script can take a TMC xyz file and convert it into a SMILES. The repository also contains the generated SMILES sets for the CSD and tmQMg datasets.

## References

[1] RDKit: Open-source cheminformatics. http://www.rdkit.org

[2] Groom CR, Bruno IJ, Lightfoot MP, Ward SC (2016) The Cambridge structural database. Acta Crystallogr Sect B Struct Sci Cryst Eng Mater 72:171–179 10.1107/S2052520616003954 PMC4822653 27048719

[3] Vela S, Laplaza R, Cho Y, Corminboeuf C (2022) cell2mol: encoding chemistry to interpret crystallographic data, NPJ Comput Mater 8: 1–8

[4] Balcells D, Skjelstad BB (2020) TmQM dataset-quantum geometries and properties of 86k transition metal complexes. J Chem Inf Model 60:6135–6146 33166143 10.1021/acs.jcim.0c01041 PMC7768608

[5] Kneiding H, Lukin R, Lang L, Reine S, Pedersen TB, De Bin R, Balcells D (2023) Deep learning metal complex properties with natural quantum graphs. Digit Discov 2:618–633

[6] Kneiding H, Nova A, Balcells D (2024) Directional multiobjective optimization of metal complexes at the billion-system scale. Nat Comput Sci 4:263–273 38553635 10.1038/s43588-024-00616-5

[7] Jensen JH (2021) xyz2mol. https://github.com/jensengroup/xyz2mol

[8] Hoffmann R (1963) An extended Hückel theory. I. hydrocarbons. J Chem Phys 39:1397–1412

[9] Rogers D, Hahn M (2010) Extended-connectivity fingerprints. J Chem Inf Model 50:742–754 20426451 10.1021/ci100050t

[10] Tom G, Hickman RJ, Zinzuwadia A, Mohajeri A, Sanchez-Lengeling B, Aspuru-Guzik A (2023) Calibration and generalizability of probabilistic models on low-data chemical datasets with DIONYSUS. Digit Discov 2:759–774

[11] O’Boyle NM, Banck M, James CA, Morley C, Vandermeersch T, Hutchison GR (2011) Open Babel: an open chemical toolbox. J Cheminform 3:33 21982300 10.1186/1758-2946-3-33 PMC3198950

[12] Frei A, Orsi M (2024) ELECTRUM: an electron configuration-based universal metal fingerprint for transition metal compounds. *ChemRxiv*10.1039/d5dd00145e PMC12548721 41142915

[13] Zhong S, Guan X (2023) Count-based Morgan fingerprint: a more efficient and interpretable molecular representation in developing machine learning-based predictive regression models for water contaminants’ activities and properties. Environ Sci Technol 57:18193–18202 37406199 10.1021/acs.est.3c02198

[14] How to turn a SMILES string into a molecular graph for pytorch geometric. https://www.blopig.com/blog/2022/02/how-to-turn-a-smiles-string-into-a-molecular-graph-for-pytorch-geometric. Accessed 12 Nov 2024.

[15] Ke G, Meng Q, Finley T, Wang T, Chen W, Ma W, Ye Q, Liu T-Y (2017) LightGBM: a highly efficient Gradient Boosting Decision Tree. Proceedings of the 31st international conference on neural information processing systems. 30:3146–3154

[16] Gilmer J, Schoenholz SS, Riley PF, Vinyals O (2017) Neural message passing for quantum chemistry, G. E. Dahl in International Conference on Machine Learning, PMLR, pp. 1263–1272

[17] Ellis JE (2006) Adventures with substances containing metals in negative oxidation states. Inorg Chem. 45:3167–3186 16602773 10.1021/ic052110i

[18] Yu HS, Truhlar DG (2016) Oxidation state 10 exists. Angewandte Chem (International ed. in English) 55: 9004–9006 10.1002/anie.201604670 27273799

[19] Strandgaard M, Seumer J, Benediktsson B, Bhowmik A, Vegge T, Jensen JH (2023) Genetic algorithm-based re-optimization of the Schrock catalyst for dinitrogen fixation. PeerJ Phys Chem 5:e30

[20] Strandgaard M, Seumer J, Jensen JH (2024) Discovery of molybdenum based nitrogen fixation catalysts with genetic algorithms. Chem Sci (R Soc Chem: 2010). 15: 10638–10650 10.1039/d4sc02227k PMC11234868 38994422

[21] Seumer J, Jensen J (2024) Beyond predefined ligand libraries: a genetic algorithm approach for de novo discovery of catalysts for the Suzuki coupling reactions. ChemRxiv

[22] Gao W, Coley CW (2020) The synthesizability of molecules proposed by generative models. J Chem Inf Model 60:5714–5723 32250616 10.1021/acs.jcim.0c00174

[23] Ertl P, Schuffenhauer A (2009) Estimation of synthetic accessibility score of drug-like molecules based on molecular complexity and fragment contributions. J Cheminform 1:8 20298526 10.1186/1758-2946-1-8 PMC3225829

[24] Kerstjens A, De Winter H (2024) Molecule auto-correction to facilitate molecular design. J Comput Aid Mol Des 38:10 10.1007/s10822-024-00549-1 PMC10873457 38363377

