# Supplementary material for: SMILES all around: structure to SMILES conversion for transition metal complexes
Source: J Cheminform. 2025 Apr 28;17:63. doi: 10.1186/s13321-025-01008-1 (PMC12039060; doi:10.1186/s13321-025-01008-1)
Supplement: Supplementary file 1 — Supplementary Material 1. [file 13321_2025_1008_MOESM1_ESM.pdf]

Supporting Information

S1 ML details

The code for the ML training is found at: github.com/jensengroup/xyz2mol_tm/tree/main/ML. The performance on the training set is shown in Figure S1.

Important hyperparameters used for all models are defined in the following.

Random Forest

- Number of estimators : 200
- Minimum samples per leaf: 2

FF-NN

- 1 hidden layer with dimension 256
- Dropout after hidden layer ($p = 0.2$)

LightGBM

For the LightGBM model we use the Optuna hyperparameter optimization framework [1]. For each of the three prediction targets we optimize the hyperparameters for the LightGBM model using Optuna. The resulting hyperparameters are given in three parameter files at : github.com/jensengroup/xyz2mol_tm/tree/main/ML/lgbm_hyperparameters.

GCNN

- 300 hidden nodes
- 6 layers
- 150 epochs

Gilmer

- 256 hidden nodes
- 6 layers
- 300 epochs

tmQMg models

For the Baseline, u-NatQG, and d-NatQG models we use the same hyperparameters and training procedure as Kneiding et al. [2].

S2 Comparing SMILES

Table 1 in the main text compared SMILES sets for the tmQMg dataset. Here we expand on some aspects of this comparison. When comparing SMILES, we use a timeout of 30 seconds on resonance check. Therefore a small subset of the comparisons fail due to the timeout and therefore contributes to the non-equal percentage. For the tmQMg SMILES comparisons the number of timeout were around 20-100. Therefore, the contribution is negligible.

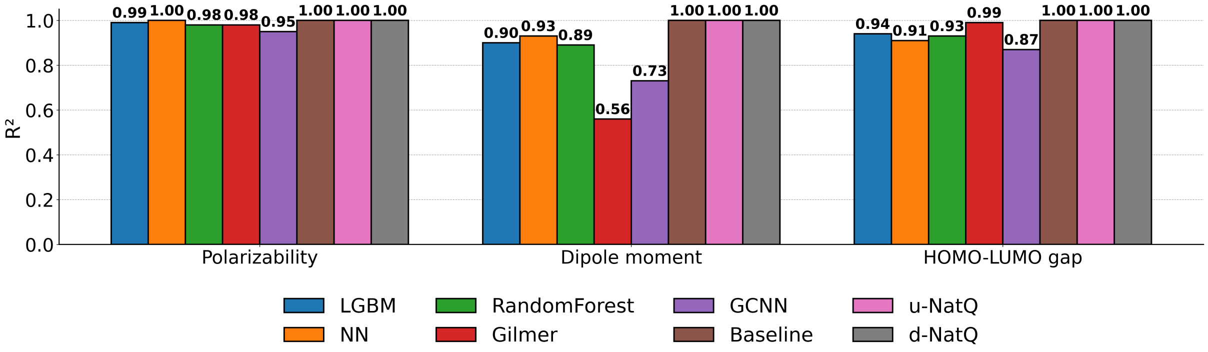

Figure S1: Comparison of R^2 values for the 5 SMILES based models versus the 3 NBO-DFT based models for the training set.

The comparison in Table 1 was done between CSD SMILES and DFT based SMILES. We perform the same comparison between the CSD SMILES and the CSD based Hückel SMILES and show the results in Table S1. It is clear that using the CSD structures directly yields a higher similarity. This is mainly attributed to the fact that we avoid potential coordination issues as bonds could be broken or formed during DFT optimization. For the **Resonance TMC** comparison there is an increase of ≈ 6000 equal SMILES when using the CSD structures.

**Method**	**SMILES pair**	
	CSD/Hückel(CSD)	CSD/Hückel(DFT)
**Direct comparison**	40 837/53 168	38 073/53 167
**Resonance TMC**	44 430/53 168	38 442/53 167
**Disconnect-Resonance**	41 840/53 168	45 734/53 167

Table S1: Number of equal SMILES when comparing SMILES pairs with different methods. **Direct comparison** compares the canonical SMILES, **Resonance TMC** compares the SMILES resonance sets and **Disconnect-Resonance** compares the resonances of ligands when disconnected from the TM.

.

S3 Analyzing SMILES

We provide two jupyter notebooks that analyzes different aspects of the SMILES sets. The first illustrates how to get the numbers in Table 1.

`github.com/jensengroup/xyz2mol_tm/comparing_smiles/comparing_smiles.ipynb`,

The second highlights important differences and things to be aware of with the 3 SMILES sets.

`github.com/jensengroup/xyz2mol_tm/comparing_smiles/highlight_smiles_problems.ipynb`,

Since the creation of tmQMg, 7 structures have been deleted from the database. They are therefore marked in the large CSD set as `not_in_database` and have no corresponding SMILES.

S3.1 Examples

Two examples of the Hückel and NBO smiles differing by 2 charge units are shown in Figure S2. For **ACTBPD**, the flexibility in charge assignment of the Hückel method means the carbon/sulphur coordinating ligand is assigned a charge of 1+ which results in a neutral Pd. The NBO on the other hand has found a ligand charge of 1- which results in a Pd^{2+}. For **AHUDIR** we have the opposite where Hückel determines the ligand with the haptic ring to have a charge of -2 and NBO determines it to a charge of 0.

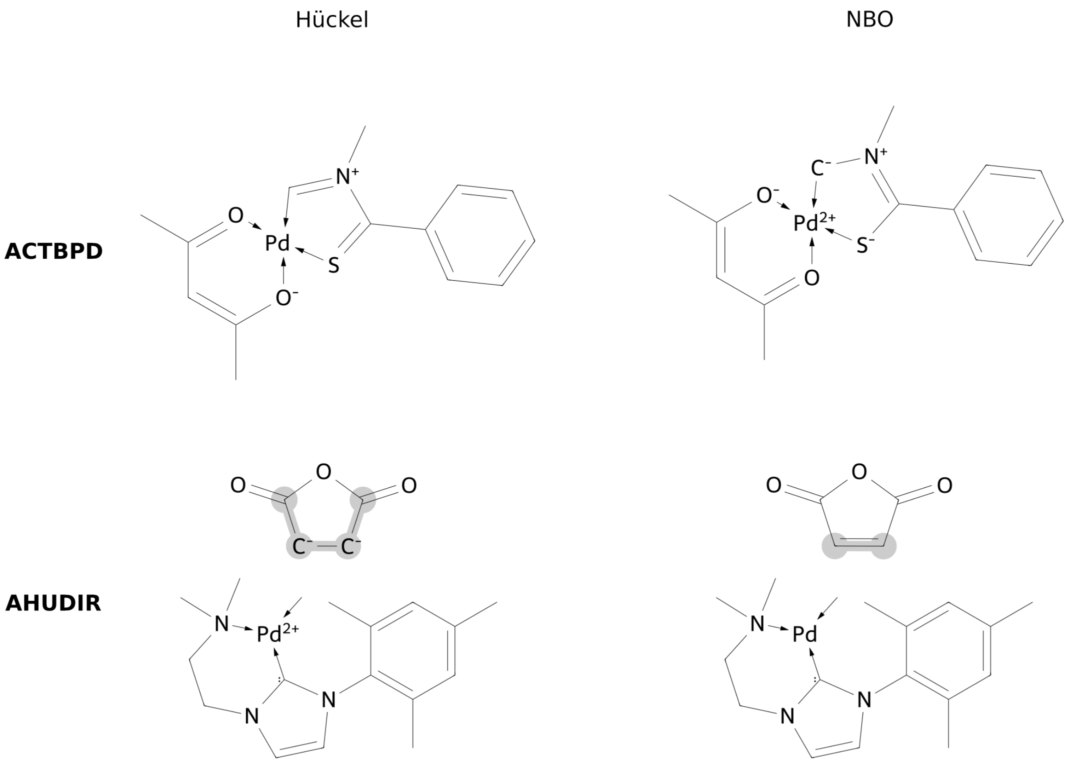

Figure S2: Two examples of the Hückel and NBO SMILES assigning different charges to ligands. Both cases lead to different Pd oxidation states.

Figure 7a in the main text illustrated the fact that some SMILES had TMCs with extreme oxidation states and here these are analyzed further. Of the 59 878 huckel SMILES, 160 have oxidation states below 0 (0.2%) and 26 have oxidation states above 10 (0.06%). The fraction of these edge cases is therefore very low. Figure S3 illustrates a case where Hg is assigned an oxidation state of 14+ with the Hückel method. It is clear from the molecular graph that the high oxidation state comes from the multiple O fragments. In contrast, the fully connected graph for the CSD SMILES on the right seems like the more accurate representation. In the Hückel method a chloride is only allowed to have a single bond. Since that is not the case here, the bonds to the TM is broken which results in multiple fragments containing Cl and O. It is therefore a result of a design choice that we get these edge cases for the Hückel SMILES. Almost all of the 26 cases with ¿ +10 TM oxidation state comes from this chloride constraint.

Figure S4 shows an example of two SMILES where the TM has negative oxidation states. The overall charge of both complexes is -1. For **ALOBIM** the naive approach where we get the charge of the TM atom will give you an oxidation state of -1. However, due to the nature of the hydride ligand that is not correct. In fact, the negative charge belongs to the hydride and the TM has an oxidation state of 0. RDKit will in some cases remove a datively bound hydride and instead put it as an implicit hydrogen on the TM.

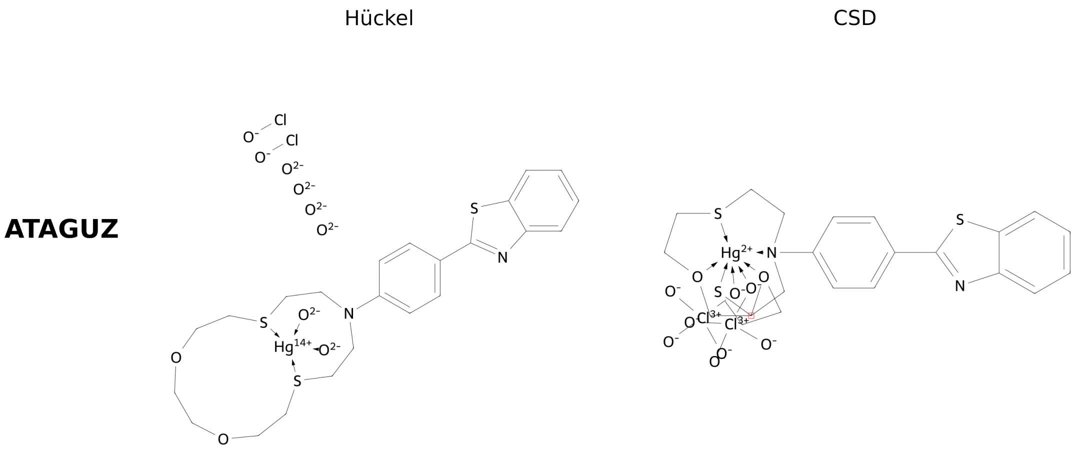

Figure S3: Example of a SMILES with an unusually high oxidation state on the TM atom.

Therefore, this implicit hydrogen with a negative charge has to be taken into account when calculating the TM oxidation state. An example of this is given in a notebook in the repo : https://github.com/jensengroup/xyz2mol_tm/blob/main/comparing_smiles/highlight_smiles_problems.ipynb.

On the other hand we have **ACIFOJ** with a negative overall charge that comes from Au. However, we see that the solution found for the two identical ligands is neutral. Another possible solution could have been -2 with the two negative charges on the sulfur. In that case the oxidation state of Au would have been +3 which is a common oxidation state for Au. Handling this in a consistent manner is complicated as one has to consider multiple factors. One needs to consider which coordination and TM atoms are involved and potentially how to handle heteroleptic environments.

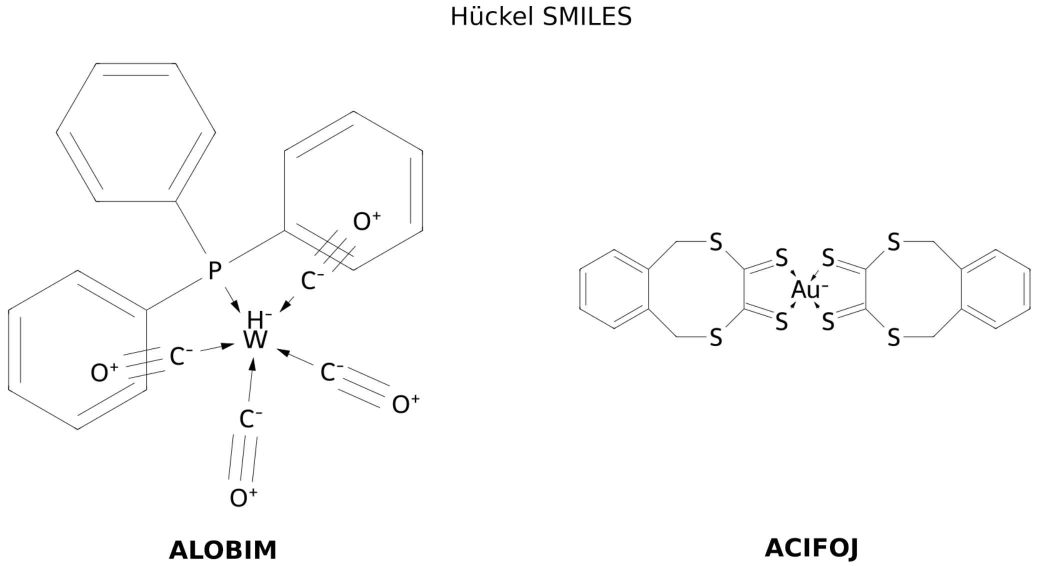

Figure S4: Two examples of the Hückel SMILES with negative oxidation states.

S4 Stereochemistry of metal center

It is possible to extract TM stereochemistry from an RDKit mol object if the 3D information for a TMC is present. Then one can use the `AssignAtomChiralTagsFromStructure` functionality to get the TMC geometry and ligand arrangement around the TMC. However, the functionality in RDKit (2024.03.3) does not seem to correctly extract this information. As a result, the connection between the extracted geometry tag and the actual 3D geometry often needs to be questioned. Additionally, the ligand arrangement around the central TM appears to have a random element which makes the tag not trustworthy. We have submitted a bug report going through the details of the issue: https://github.com/rdkit/rdkit/issues/7948.

References

[1] T. Akiba, S. Sano, T. Yanase, T. Ohta, M. Koyama, *CoRR* **2019**, *abs/1907.10902*.

[2] H. Kneiding, R. Lukin, L. Lang, S. Reine, T. B. Pedersen, R. De Bin, D. Balcells, *Digital Discovery* **2023**, *2*, 618–633.
